# Supplementary material for: Different miRNA expression profiles between human breast cancer tumors and serum
Source: Front Genet. 2014 May 27;5:149. doi: 10.3389/fgene.2014.00149 (PMC4033838; doi:10.3389/fgene.2014.00149)
Supplement: Supplementary file 1 [file DataSheet1.DOCX]

**Table S1. Number of reads from small RNA-seq**

| Sample | Total Reads | Uniquely mapped reads | Percentage of Uniquely mapped (%) |
| --- | --- | --- | --- |
| Serum from BC patient | 9538940 | 3494623 | 36.64% |
| Serum from healthy volunteers | 9752317 | 2947601 | 30.22% |
| BC patient tissue | 7773017 | 840205 | 10.81% |
| Tumor Adjacent Tissue | 7309040 | 1752925 | 23.98% |

Note: ≤2 mismatches are allowed.

**Table S2. Number of the differentially expressed miRNAs in breast cancer**

| Sample | Up-regulated genes Number | Down-regulated genes Number |
| --- | --- | --- |
| Serum^a^ | 18 | 91 |
| Tissue^b^ | 120 | 54 |
| Overlap | 3 | 7 |

^a^: breast cancer tumors versus adjacent normal tissues; ^b^:breast cancer serum versus serum from healthy individuals.

**Table S3. The potential miRNA-target interactions which may play an important role in breast cancer**

| miRNA | Gene Symbol | Gene Description | Reference NO^a^ | | Expression^b^ |
| --- | --- | --- | --- | --- | --- |
| miR-598 | AMIGO2 | Adhesion molecule with Ig-like domain 2 | | 6 | down |
| miR-598 | ZNF395 | Zinc finger protein 395 | | 7 | down |
| miR-598 | EEPD1 | Endonuclease/exonuclease/phosphatase family domain containing 1 | | 5 | down |
| miR-598 | KLK10 | Kallikrein-related peptidase 10 | | 6 | down |
| miR-598 | KREMEN1 | Kringle containing transmembrane protein 1 | | 5 | down |
| miR-598 | MSI2 | Musashi homolog 2 (Drosophila) | | 7 | up |
| miR-598 | NTN4 | Netrin 4 | | 7 | down |
| miR-598 | PPL | Periplakin | | 5 | down |
| miR-598 | PTGER3 | Prostaglandin E receptor 3 (subtype EP3) | | 5 | down |
| miR-598 | THBS2 | Thrombospondin 2 | | 5 | up |
| miR-598 | ZC3H6 | Zinc finger CCCH-type containing 6 | | 5 | down |
| miR-485-5p | AMOTL2 | Angiomotin like 2 | | 5 | down |
| miR-485-5p | NOVA1 | Neuro-oncological ventral antigen 1 | | 6 | down |
| miR-485-5p | CHD9 | Chromodomain helicase DNA binding protein 9 | | 8 | down |
| miR-485-5p | CRY2 | Cryptochrome 2 (photolyase-like) | | 5 | down |
| miR-485-5p | GOLM1 | Golgi membrane protein 1 | | 6 | up |
| miR-485-5p | RBPMS | RNA binding protein with multiple splicing | | 11 | down |
| miR-485-5p | SAMD9 | Sterile alpha motif domain containing 9 | | 7 | up |
| miR-485-5p | SLC22A23 | Solute carrier family 22, member 23 | | 5 | up |
| miR-485-5p | CD59 | CD59 molecule, complement regulatory protein | | 7 | down |
| miR-382 | AMOTL2 | Angiomotin like 2 | | 5 | down |
| miR-382 | CABC1 | Chaperone, ABC1 activity of bc1 complex homolog (S. Pombe) | | 7 | down |
| miR-382 | CHST9 | Carbohydrate (N-acetylgalactosamine 4-0) sulfotransferase 9 | | 6 | down |
| miR-382 | EEF1A1 | Eukaryotic translation elongation factor 1 alpha 1 | | 6 | down |
| miR-382 | GREM2 | Gremlin 2, cysteine knot superfamily, homolog (Xenopus laevis) | | 6 | down |
| miR-382 | MATN2 | Matrilin 2 | | 7 | down |
| miR-382 | MID1 | Midline 1 (Opitz/BBB syndrome) | | 10 | down |
| miR-382 | NFIA | Nuclear factor I/A | | 8 | down |
| miR-382 | NFIB | Nuclear factor I/B | | 11 | down |
| miR-382 | SOX7 | SRY (sex determining region Y)-box 7 | | 7 | down |
| miR-382 | TMEM49 | Transmembrane protein 49 | | 5 | up |
| miR-382 | ZNF587 | Zinc finger protein 587 | | 5 | up |
| miR-34c-5p | ACSL4 | Acyl-coa synthetase long-chain family member 4 | | 6 | down |
| miR-34c-5p | MET | Met proto-oncogene (hepatocyte growth factor receptor) | | 7 | down |
| miR-34c-5p | ACSL1 | Acyl-coa synthetase long-chain family member 1 | | 6 | down |
| miR-34c-5p | ARID4B | AT rich interactive domain 4B (RBP1-like) | | 5 | up |
| miR-34c-5p | CAPN6 | Calpain 6 | | 6 | down |
| miR-34c-5p | COL12A1 | Collagen, type XII, alpha 1 | | 8 | up |
| miR-34c-5p | DLL1 | Delta-like 1 (Drosophila) | | 5 | down |
| miR-34c-5p | FUT8 | Fucosyltransferase 8 (alpha (1,6) fucosyltransferase) | | 5 | up |
| miR-34c-5p | LEF1 | Lymphoid enhancer-binding factor 1 | | 7 | up |
| miR-34c-5p | NAV1 | Neuron navigator 1 | | 5 | up |
| miR-34c-5p | NRN1 | Neuritin 1 | | 6 | down |
| miR-34c-5p | PDGFRA | Platelet-derived growth factor receptor, alpha polypeptide | | 7 | down |
| miR-34c-5p | PLCB1 | Phospholipase C, beta 1 (phosphoinositide-specific) | | 6 | down |
| miR-34c-5p | STAC2 | SH3 and cysteine rich domain 2 | | 5 | down |
| miR-34c-5p | ZNF281 | Zinc finger protein 281 | | 5 | up |
| miR-323-3p | ZNF395 | Zinc finger protein 395 | | 7 | down |
| miR-323-3p | ASPN | Asporin | | 9 | up |
| miR-323-3p | EGR3 | Early growth response 3 | | 6 | down |
| miR-323-3p | FANCI | Fanconi anemia, complementation group I | | 7 | up |
| miR-323-3p | FZD7 | Frizzled homolog 7 (Drosophila) | | 8 | down |
| miR-323-3p | JAM2 | Junctional adhesion molecule 2 | | 7 | down |
| miR-323-3p | KLF5 | Kruppel-like factor 5 (intestinal) | | 5 | down |
| miR-323-3p | PDE4B | Phosphodiesterase 4B, camp-specific (phosphodiesterase E4 dunce homolog, Drosophila) | | 5 | down |
| miR-323-3p | SEMA6D | Sema domain, transmembrane domain (TM), and cytoplasmic domain, (semaphorin) 6D | | 9 | down |
| miR-323-3p | STAT5B | Signal transducer and activator of transcription 5B | | 8 | down |
| miR-323-3p | TGFA | Transforming growth factor, alpha | | 6 | down |
| miR-323-3p | UHMK1 | U2AF homology motif (UHM) kinase 1 | | 5 | up |
| miR-323-3p | VSNL1 | Visinin-like 1 | | 5 | down |
| miR-224 | AMOTL2 | Angiomotin like 2 | | 5 | down |
| miR-224 | ACSL4 | Acyl-coa synthetase long-chain family member 4 | | 6 | down |
| miR-224 | AMIGO2 | Adhesion molecule with Ig-like domain 2 | | 6 | down |
| miR-224 | PPAP2B | Phosphatidic acid phosphatase type 2B | | 10 | down |
| miR-224 | DPYSL2 | Dihydropyrimidinase-like 2 | | 11 | down |
| miR-224 | FAM49B | Family with sequence similarity 49, member B | | 8 | up |
| miR-224 | H3F3B | H3 histone, family 3B (H3.3B) | | 8 | down |
| miR-224 | HSPA12A | Heat shock 70kda protein 12A | | 6 | down |
| miR-224 | KCTD12 | Potassium channel tetramerisation domain containing 12 | | 6 | down |
| miR-224 | MAP1B | Microtubule-associated protein 1B | | 5 | down |
| miR-224 | PPP2R1B | Protein phosphatase 2, regulatory subunit A, beta | | 6 | down |
| miR-224 | PTX3 | Pentraxin 3, long | | 7 | down |
| miR-224 | RUNX2 | Runt-related transcription factor 2 | | 5 | up |
| miR-224 | SLC4A4 | Solute carrier family 4, sodium bicarbonate cotransporter, member 4 | | 5 | down |
| miR-184 | CXCL12 | Chemokine (C-X-C motif) ligand 12 | | 5 | down |
| miR-184 | DIO2 | Deiodinase, iodothyronine, type II | | 5 | up |
| miR-184 | PPAP2B | Phosphatidic acid phosphatase type 2B | | 10 | down |
| miR-184 | ADRA2A | Adrenergic, alpha-2A-, receptor | | 8 | down |
| miR-184 | CREB3L1 | Camp responsive element binding protein 3-like 1 | | 5 | up |
| miR-184 | CREB5 | Camp responsive element binding protein 5 | | 5 | down |
| miR-184 | IGF1R | Insulin-like growth factor 1 receptor | | 5 | down |
| miR-184 | MGLL | Monoglyceride lipase | | 7 | down |
| miR-184 | PKP1 | Plakophilin 1 (ectodermal dysplasia/skin fragility syndrome) | | 5 | down |
| miR-184 | PPARA | Peroxisome proliferator-activated receptor alpha | | 8 | down |
| miR-184 | RUNX1 | Runt-related transcription factor 1 | | 5 | up |
| miR-184 | SLC25A25 | Solute carrier family 25 (mitochondrial carrier; phosphate carrier), member 25 | | 6 | down |
| miR-184 | SLC7A2 | Solute carrier family 7 (cationic amino acid transporter, y+ system), member 2 | | 5 | down/up^c^ |
| miR-184 | THBD | Thrombomodulin | | 6 | down |
| miR-132 | NOVA1 | Neuro-oncological ventral antigen 1 | | 6 | down |
| miR-132 | C2orf55 | Chromosome 2 open reading frame 55 | | 5 | up |
| miR-132 | C5orf13 | Chromosome 5 open reading frame 13 | | 9 | up |
| miR-132 | CALU | Calumenin | | 5 | up |
| miR-132 | DAZAP2 | DAZ associated protein 2 | | 5 | up |
| miR-132 | DPYSL3 | Dihydropyrimidinase-like 3 | | 5 | down |
| miR-132 | HBEGF | Heparin-binding EGF-like growth factor | | 6 | down |
| miR-132 | HN1 | Hematological and neurological expressed 1 | | 7 | up |
| miR-132 | OLFM1 | Olfactomedin 1 | | 7 | down |
| miR-132 | PRKD1 | Protein kinase D1 | | 7 | down |
| miR-132 | PSMD12 | Proteasome (prosome, macropain) 26S subunit, non-atpase, 12 | | 5 | up |
| miR-132 | SEMA6A | Sema domain, transmembrane domain (TM), and cytoplasmic domain, (semaphorin) 6A | | 10 | down |
| miR-132 | SOX4 | SRY (sex determining region Y)-box 4 | | 6 | up |
| miR-132 | SPRY1 | Sprouty homolog 1, antagonist of FGF signaling (Drosophila) | | 7 | down |
| miR-132 | TCF7L1 | Transcription factor 7-like 1 (T-cell specific, HMG-box) | | 6 | down |
| miR-132 | TLN2 | Talin 2 | | 7 | down |
| miR-132 | TRIM2 | Tripartite motif-containing 2 | | 6 | down |
| miR-125b | CBX7 | Chromobox homolog 7 | | 7 | down |
| miR-125b | LBH | Limb bud and heart development homolog (mouse) | | 5 | down |
| miR-125b | OLFML2A | Olfactomedin-like 2A | | 6 | down |
| miR-125b | PELI2 | Pellino homolog 2 (Drosophila) | | 5 | down |
| miR-125b | STARD13 | Star-related lipid transfer (START) domain containing 13 | | 5 | down |
| miR-1246 | CD59 | CD59 molecule, complement regulatory protein | | 7 | down |
| miR-1246 | CXCL12 | Chemokine (C-X-C motif) ligand 12 | | 5 | down |
| miR-1246 | DIO2 | Deiodinase, iodothyronine, type II | | 5 | up |
| miR-1246 | MET | Met proto-oncogene (hepatocyte growth factor receptor) | | 7 | down |
| miR-1246 | ADH1B | Alcohol dehydrogenase 1B (class I), beta polypeptide | | 10 | down |
| miR-1246 | ALDH1A3 | Aldehyde dehydrogenase 1 family, member A3 | | 8 | down |
| miR-1246 | COL5A1 | Collagen, type V, alpha 1 | | 9 | up |
| miR-1246 | CTSK | Cathepsin K | | 5 | down/up |
| miR-1246 | CYP24A1 | Cytochrome P450, family 24, subfamily A, polypeptide 1 | | 9 | down |
| miR-1246 | DCX | Doublecortin | | 5 | down |
| miR-1246 | DIO1 | Deiodinase, iodothyronine, type I | | 8 | up |
| miR-1246 | FGFR2 | Fibroblast growth factor receptor 2 | | 7 | down |
| miR-1246 | GLRB | Glycine receptor, beta | | 6 | down |
| miR-1246 | IGF2 | Insulin-like growth factor 2 (somatomedin A) | | 6 | down |
| miR-1246 | IGFBP5 | Insulin-like growth factor binding protein 5 | | 5 | down |
| miR-1246 | ITGB2 | Integrin, beta 2 (complement component 3 receptor 3 and 4 subunit) | | 7 | up |
| miR-1246 | LEP | Leptin | | 6 | down |
| miR-1246 | PECAM1 | Platelet/endothelial cell adhesion molecule | | 6 | down |
| miR-1246 | PTCH1 | Patched homolog 1 (Drosophila) | | 6 | down |
| miR-1246 | THRB | Thyroid hormone receptor, beta (erythroblastic leukemia viral (v-erb-a) oncogene homolog 2, avian) | | 5 | down |
| miR-1246 | VDR | Vitamin D (1,25- dihydroxyvitamin D3) receptor | | 5 | up |

^a^: The number of breast cancer published studies which support the target genes differential expression; ^b^: The Expression change recorded in oncomine database; down/up ^c^ means there are 5 studies supporting over-expression of this gene, and 5 studied supporting down-expression of this gene.
